# Trauma as a mediator of childhood adversity and mental illness in South Africa: A path analysis

**DOI:** 10.4102/sajpsychiatry.v31i0.2276

**Published:** 2025-05-20

**Authors:** Michael D. Galvin, Ann Scheunemann, Lesley Chiwaye, Zoleka Luvuno, Andrew W. Kim, Aneesa Moolla

**Affiliations:** 1 Health Economics and Epidemiology Research Office (HE^2^RO), University of the Witwatersrand, Johannesburg, South Africa

**Keywords:** South Africa, Johannesburg, ACEs, trauma, mental health

## Abstract

**Background:**

South Africa bears a high burden of adverse childhood events (ACEs), which have been identified as a primary factor that can lead to negative mental health outcomes for adults. While studies within South Africa have examined the associations between ACEs, adult trauma and adult mental illness, there is less knowledge of how these preceding factors interact to affect mental distress together and which ACEs are most likely to lead to adverse mental health outcomes.

**Aim:**

The main aim of this study was to explore the mediating effects of recent adult trauma on mental illness among patients at two psychiatric hospitals in Johannesburg, South Africa, using path analysis.

**Setting:**

This study took place at two public psychiatric facilities in Johannesburg, South Africa.

**Methods:**

Surveys were conducted with 309 adults living in Gauteng province. Mediational path analysis explored the association between ACEs, adult traumatic events, and depression, anxiety, and stress.

**Results:**

Adult traumatic events partially mediated the association between verbal abuse, emotional neglect, mental illness and substance use in the household as a child and adult mental illness. Adult traumatic events fully mediated the associations between experiencing domestic violence in childhood or child sexual abuse.

**Conclusion:**

This study highlights the importance of disaggregating ACEs when exploring their effects while also reinforcing previous findings that ACEs increase the likelihood of experiencing adult trauma and adult mental illness.

**Contribution:**

Future studies should further pinpoint which ACEs are most impactful and target those for prevention in childhood and intervention in adulthood to mitigate their deleterious impacts.

## Introduction

Globally there are significant variations in exposure to trauma;^1^ however, the high rate of post-traumatic stress in sub-Saharan Africa indicates a high prevalence in the region.^2^ Recent meta-analyses suggest that children in sub-Saharan Africa have higher rates of sexual, physical and emotional abuse than in other regions of the world.^3^ Coupled with the world’s highest rates of poverty, poor housing, increasing urbanisation and insecurity, sub-Saharan Africa is today considered a region in which rates of trauma are rising the fastest and are expected to continue in this direction.^4^

Within South Africa, this has prompted an increase in research on trauma exposure in recent years. One study with a clinical population in South Africa found a 75% prevalence rate of past trauma exposure, with a 43.8% rate of multiple traumatic events.^5^ Another recently published study compared clinical and non-clinical populations and found high rates of exposure to trauma in both groups, although significantly more clinical participants were exposed to more than four traumatic events.^6^ Traumatic events commonly found within South Africa include the traumatic death or injury of a loved one, physical assault, sexual assault and witnessing violence.

In South Africa, mental illness as a result of traumatic experiences has been declared a national public health concern because of particular high rates of violence, crime and serious accidents.^7^ Studies have examined how these trends have resulted in particularly high rates of traumatic exposure among South Africans.^8,9,10^ This has been seen to lead to serious consequences for mental health on a national scale.

Mental illness is an increasing concern in South Africa, where an estimated 92% of people who need care for psychiatric disorders are reportedly not receiving any biomedical treatment.^11^ Many of these people living with serious mental illness are suffering because of exposure to trauma, either during childhood or adulthood.^12^ Globally, several systematic reviews have found that childhood abuse predicts anxiety and depression later in life.^13,14^ Similarly, the literature also confirms that multiple traumatic events in adulthood are also associated with greater psychiatric symptoms.^15,16,17^ In South Africa as well, mental disorders such as post-traumatic stress disorder (PTSD), major depression and anxiety are considered psychological sequelae of trauma, and studies have documented how histories of interpersonal trauma among South Africans are also associated with a higher risk of mental disorders.^10,18,19,20,21^ Recent studies have also begun to examine how childhood adversity along with recent stressors and trauma in adulthood can interact to increase the risk of mental disorders among adults.^9,22,23^

While the literature has established a firm link between childhood adversity and adult mental health,^24,25,26^ few of these studies have focused on sub-Saharan Africa. This suggests a significant gap in the existing literature understanding the psychological burden of childhood adversity globally. Adverse childhood experiences (ACEs) – or stressful experiences during childhood – have been found to occur at high rates in South Africa. Studies have found that symptoms of post-traumatic stress in children in South Africa may reach up to 22%, with significantly higher rates among females.^9,27^ These ACEs have been shown to have negative effects on mental health both in childhood and adulthood among South Africans.^28^

Most studies with ACEs explore the impact of aggregated scores on various outcomes; therefore, very little is known about how specific types of adversity in childhood may relate to adult well-being.^29^ In assessing cumulative ACE scores, each adverse experience is weighted the same and it is therefore not possible to determine if certain experiences are more impactful than others on mental health outcomes like stress, depression and anxiety. One recent study determined that adults who experienced family mental illness or sexual abuse in childhood were more likely to be diagnosed with depression.^30^ This study, however, was conducted in the United States, and the results may not translate to the South African context. To our knowledge, no ACE studies conducted in South Africa to date have explored disaggregated ACE domains. Given the prevalence of childhood adversity in the country and the relationship between ACEs and poor mental health in adulthood, a better understanding of the extended impacts of individual ACEs is warranted.

Previous research with this study sample has found that both ACEs and adult traumatic events were associated with significant increases in anxiety, depression and severe stress.^31^ This study aimed to go further by: (1) examining *disaggregated* ACEs as a predictor of depression, anxiety and stress symptoms, and (2) exploring whether the impact of childhood trauma on these symptoms was *mediated* by adult traumatic events. Findings from this study may help guide the development of interventions that are targeted to treat and prevent these symptoms and which are based on specific traumas experienced during childhood, thereby optimising mental health outcomes through increased programmatic efficacy.

## Research methods and design

### Sample

Sampling, procedures and measures were described in a previous publication.^31^ Data for this cross-sectional study were collected between January and July 2022 at two public psychiatric facilities in Johannesburg, South Africa – Helen Joseph Hospital and Alexandra 18th Avenue Clinic. The study assessed the perceptions and experiences of mental illness and treatment among psychiatric patients at these locations. A convenience sampling technique was used to recruit participants receiving psychiatric care at these two hospitals in Johannesburg, South Africa (*n* = 309). All patients who identified as being of Black/African descent, aged 18 years or above, and able to provide written informed consent were eligible to participate in the study. Individuals who did not meet the inclusion criteria or self-reported having a serious mental illness or disability that would prevent them from participating in the survey were excluded from the study.

### Measures

Demographics: Participants reported their age, sex, psychiatric history, history of mental illness in family members, income, marital status, highest level of education achieved and number of people living in the household.

Adverse childhood experiences were measured with the 10-item version of the ACEs – International Questionnaire.^32^ In this study, all items on the instrument were binary variables. The presence or absence of each ACE was assessed individually to determine its association with adult trauma and mental health.

Stress was measured using the 10-item Perceived Stress Scale (PSS).^33^ Participants were asked to weigh their stressful life experiences in the past month. The scale has been previously used in research in South Africa.^33^

Traumatic events were measured using a 12-item binary (yes/no) index assessing the experience of traumatic events (homelessness, rape, injury, family separation, natural disaster, murder, domestic violence and serious automobile accident). A high index score indicates a larger number of traumatic events experienced by the participant.

Anxiety symptoms were assessed using the Generalized Anxiety Disorder (GAD-7) screen tool. The measure consists of seven Likert scale items that have been validated for assessing GAD in clinical and research environments in different cultural settings.^34^ The seven-item measure assessed the severity of symptoms according to reported responses with a maximum possible score of 21.^35^

Depressive severity was assessed using the Patient Health Questionnaire 9, which includes nine items.^34,36,37^ The scale consists of nine items scored on a three-point Likert scale ranging from 0 (not at all) to 3 (nearly every day).

### Data analysis

Data were analysed using R statistical software 4.2.2. Descriptive data were obtained for the sample (Table 1), and internal consistency of the measures of depression, anxiety, stress, and ACEs were determined using Cronbach’s alpha. Diagonally weighted least squares path analysis with *lavaan*^38^ was used to assess aggregated traumatic events in adulthood as a mediator between individual ACEs and depression, anxiety and stress. Goodness-of-fit for each model was assessed using χ^2^, the Tucker–Lewis Index (TLI) with a cutoff of 0.90,^39^ and the root mean square error of approximation (RMSEA) with a cutoff of 0.08.^40^ Standardised and unstandardised estimates were derived in the models so that the degree of influence could be determined.

**TABLE 1:** Descriptive statistics.

Variables	*n*	%	Mean	s.d.	Skew	Kurtosis
**Continuous**						
Age	309	-	38.52	12.50	0.39	−0.68
Total adverse childhood events	309	-	3.21	2.28	0.47	−0.58
Total adult traumatic events	309	-	2.50	1.74	0.58	−0.27
Depression	309	-	9.83	6.15	0.64	−0.44
Anxiety	309	-	7.90	4.75	0.56	−0.51
Stress	309	-	20.28	6.52	−0.11	−0.23
**Categorical**						
**Gender**						
Female	172	55.660	-	-	-	-
Male	137	44.340	-	-	-	-
**Hospital**						
Helen Joseph	162	52.430	-	-	-	-
Alexandria	147	47.570	-	-	-	-
**Education**						
No formal	4	1.290	-	-	-	-
Elementary	103	33.330	-	-	-	-
High School	140	45.310	-	-	-	-
Tertiary	62	20.060	-	-	-	-
**Physical health**						
Very good	12	3.880	-	-	-	-
Good	239	77.350	-	-	-	-
Bad	58	18.770	-	-	-	-
Very bad	0	0.000	-	-	-	-
**Alcohol use**						
Yes	123	39.815	-	-	-	-
No	186	60.190	-	-	-	-
**Substance use**						
Yes	36	11.650	-	-	-	-
No	273	88.350	-	-	-	-

s.d., standard deviation.

### Ethical considerations

Ethical clearance to conduct this study was obtained from the University of the Witwatersrand Human Research Ethics Committee (No. M210815). Participants were approached by the study lead, co-investigators and trained research assistants at the two hospitals. After written informed consent was obtained, each participant was interviewed by a trained research assistant. The interviews were conducted in person at a private location at the hospital. All potential participants were screened for eligibility in person. All participants signed and received a copy of the consent form to keep for their records and received remuneration in the amount of 100 ZAR or South African Rand (US$ 7). Data confidentiality was maintained by keeping all information on password-protected devices that were only accessible for study team members.

## Results

**FIGURE 1:**
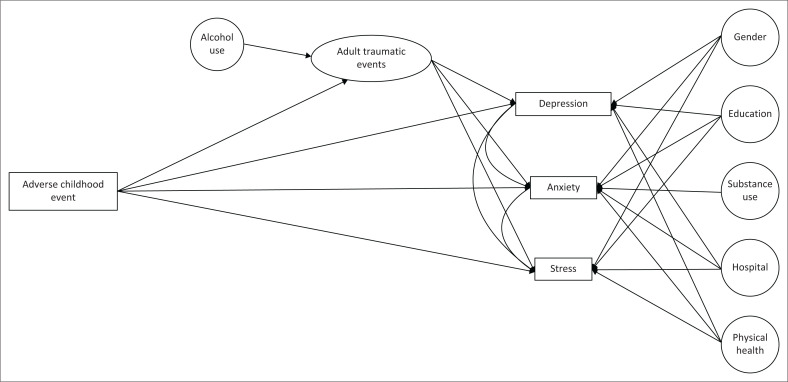
Path analysis of adult traumatic events as a mediator between adverse childhood events and adult mental illness.

The study enrolled a total of 309 participants, all of whom completed the measures in their entirety. Skewness and kurtosis of continuous data were mild but violated the Shapiro–Wilks normality test.^41^ Correlations between total adult traumatic events, total depression, total anxiety, total ACEs and total stress were mild-to-moderate (Table 2); correlations with other continuous variables were weak. Point-biserial correlations between gender and total depression (rpb = –0.25, *p* < 0.001), total anxiety (rpb = –0.21, *p* < 0.001) and total stress (rpb = –0.23, *p* < 0.001) were all significant, as was the hospital at which the participant was interviewed; correlations were rpb = –0.22, *p* < 0.001, rpb = –0.18, *p* = 0.001 and rpb = –0.19, *p* < 0.001 for depression, stress and anxiety, respectively. Additionally, substance use correlated with anxiety (rpb = –0.12, *p* = 0.03), and alcohol use correlated with the total number of adult traumatic events (rpb = –0.14, *p* = 0.02). Analysis of variances (ANOVAs) was used as a preliminary evaluation of the relationship between categorical and ordinal variables and mental health outcomes. Subjective physical health was significantly associated with depression, *F*(1, 309) = 53.24, *p* < 0.001; anxiety *F*(1, 309) = 40.60, *p* < 0.001; and stress, *F*(1, 309) = 43.06, *p* < 0.001. Additionally, education was found to be associated with depression, *F*(1, 309) = 4.79, *p* = 0.03 and anxiety, *F*(1, 309) = 6.58, *p* = 0.01. Based on these findings, gender, hospital setting, physical health and education were retained as control variables for depression, anxiety and stress in the final models. Substance use was retained as a control for anxiety, and alcohol use was retained as a control for total adult traumatic events. Additionally, depression, anxiety and stress were allowed to covary in the path analysis. The final model, used for all subsequent analyses, is depicted in Figure 1.

**TABLE 2:** Correlations between continuous variables.

Variables	Age	Monthly Income	Number of Children	Household size	Number of Rooms	Total Traumatic Events	Total Depression	Total Anxiety	Total ACEs	Total Stress
Age	1.00	-	-	-	-	-	-	-	-	-
Monthly income	−0.10	1.00	-	-	-	-	-	-	-	-
Number of children	0.50	−0.02	1.00	-	-	-	-	-	-	-
Household size	0.04	0.01	0.09	1.00	-	-	-	-	-	-
Number of rooms	0.05	0.20	−0.11	0.38	1.00	-	-	-	-	-
Total traumatic events	0.12	−0.01	0.04	0.00	0.11	1.00	-	-	-	-
Total depression	0.14	0.01	−0.10	−0.10	−0.01	0.28	1.00	-	-	-
Total anxiety	0.16	0.03	−0.11	−0.04	−0.04	0.27	0.75	1.00	-	-
Total ACEs	0.15	0.05	−0.03	−0.02	0.00	0.36	0.28	0.34	1.00	-
Total stress	0.05	−0.01	−0.02	−0.05	−0.07	0.31	0.69	0.70	0.32	1.00

ACEs, adverse childhood events.

A path analysis was conducted to evaluate total ACEs as the independent variable in the model. Total adult traumatic events were found to partially mediate the relationship between total ACEs and depression, anxiety and stress (Table 3). Based on these findings, individual ACEs were tested, to determine whether differences could be observed between them. Model fits for each respective model are detailed in Table 4. Total adult traumatic events partially mediated the associations between verbal abuse, emotional neglect and mental illness in the household as a child and all three adult mental health outcomes (Table 3). Total adult traumatic experiences partially mediated the association between experiencing substance use in the household as a child and experiencing anxiety and stress as an adult. Additionally, total adult traumatic events fully mediated the association between experiencing domestic violence in childhood and experiencing stress as an adult, and fully mediated the association between childhood sexual abuse and anxiety and stress. All other associations showed a non-significant direct effect, either between the ACE (independent variable) and the mental health outcome or between the total adult traumatic events (mediator) and the mental health outcome.

**TABLE 3:** Indirect and direct effects of the path analyses.

Variables	*B*	s.e.	*β*	*Z*	*p*	Goodness of fit indices			
						χ^2^	*df*	TLI	RMSEA [Cl]
**Total ACEs**	-	-	-	-	-	17.37	11	0.98	0.04 [0.00–0.08]
**Direct effects**									
ACEs -> Depression	0.62	0.14	0.23	4.46	< 0.001	-	-	-	-
ACEs -> Anxiety	0.59	0.11	0.29	5.56	< 0.001	-	-	-	-
ACEs -> Stress	0.78	0.16	0.28	4.87	< 0.001	-	-	-	-
ACEs -> TTE	0.26	0.04	0.34	6.01	< 0.001	-	-	Fit	-
**Indirect effects**									
ACEs -> TTE -> Depression	0.13	0.06	0.05	2.30	0.020*	-	-	*R*^2^ = 0.27^a^	-
ACEs -> TTE -> Anxiety	0.09	0.04	0.04	2.00	0.050*	-	-	*R*^2^ = 0.27^a^	-
ACEs -> TTE -> Stress	0.17	0.60	0.06	2.90	0.004*	-	-	*R*^2^ = 0.26^a^	-
**Verbal abuse**	-	-	-	-	-	19.90	11	0.98	0.05 [0.01–0.09]
**Direct effects**									
ACEs -> Depression	2.51	0.65	0.20	3.89	< 0.001	-	-	-	-
ACEs -> Anxiety	2.92	0.51	0.24	4.51	< 0.001*	-	-	-	-
ACEs -> Stress	2.66	0.74	0.20	4.87	< 0.001	-	-	-	-
ACEs -> TTE	0.70	0.20	0.20	3.42	0.001*	-	-	Fit	-
**Indirect effects**									
ACEs ->TTE -> Depression	0.42	0.18	0.03	2.31	0.020*	-	-	*R*^2^ = 0.27^a^	-
ACEs -> TTE -> Anxiety	0.32	0.14	0.03	2.19	0.030*	-	-	*R*^2^ = 0.26^a^	-
ACEs -> TTE -> Stress	0.58	0.21	0.04	2.71	0.007*	-	-	*R*^2^ = 0.24^a^	-
**Physical abuse**	-	-	-	-	-	17.65	11	0.98	0.04 [0.00–0.08]
**Direct effects**									
ACEs -> Depression	1.23	0.72	0.09	1.72	0.090	-	-	-	-
ACEs -> Anxiety	1.17	0.57	0.11	2.05	0.040*	-	-	-	-
ACEs -> Stress	1.27	0.79	0.09	1.60	0.110	-	-	-	-
ACEs -> TTE	0.46	0.21	0.12	2.13	0.030*	-	-	Fit	-
**Indirect effects**									
ACEs -> TTE -> Depression	-	-	-	-	-	-	-	-	-
ACEs -> TTE -> Anxiety	0.25	0.14	0.02	1.79	0.070	-	-	-	-
ACEs -> TTE -> Stress	-	-	-	-	-	-	-	-	-
**Sexual abuse**	-	-	-	-	-	15.11	11	0.99	0.04 [0.00–0.07]
**Direct effect**									
ACEs -> Depression	1.39	0.87	0.09	1.60	0.110	-	-	-	-
ACEs -> Anxiety	1.41	0.64	0.12	2.20	0.030*	-	-	-	-
ACEs -> Stress	2.12	0.83	0.13	2.54	0.010*	-	-	-	-
ACEs -> TTE	1.18	0.26	0.27	4.45	< 0.001*	-	-	Fit	-
**Indirect effect**									
ACEs -> TTE -> Depression	-	-	-	-	-	-	-	-	-
ACEs -> TTE -> Anxiety	0.61	0.23	0.05	2.25	0.006*	-	-	*R*^2^ = 0.23^b^	-
ACEs -> TTE -> Stress	1.04	0.33	0.06	3.10	0.002*	-	-	*R*^2^ = 0.22^b^	-
**Emotional neglect**	-	-	-	-	-	16.46	11	0.98	0.04 [0.00–0.08]
**Direct effect**									
ACEs -> Depression	2.71	0.69	0.22	3.91	< 0.001	-	-	-	-
ACEs -> Anxiety	2.86	0.52	0.29	5.49	< 0.001	-	-	-	-
ACEs -> Stress	3.35	0.75	0.25	4.50	< 0.001	-	-	-	-
ACEs ->TTE	0.72	0.21	0.20	3.40	0.001*	-	-	Fit	-
**Indirect effect**									
ACEs ->TTE -> Depression	0.43	0.19	0.03	2.30	0.020*	-	-	*R*^2^ = 0.27^a^	-
ACEs -> TTE -> Anxiety	0.31	0.14	0.03	-	0.030*	-	-	*R*^2^ = 0.28^a^	-
ACEs -> TTE -> Stress	0.57	0.22	0.04	2.66	0.010*	-	-	*R*^2^ = 0.25^a^	-
**Physical neglect**	-	-	-	-	-	18.66	11	0.98	0.05 [0.00–0.08]
**Direct effect**									
ACEs -> Depression	1.61	1.00	0.09	1.62	0.110	-	-	-	-
ACEs -> Anxiety	1.63	0.77	0.12	2.11	0.040*	-	-	-	-
ACEs -> Stress	1.89	1.12	0.10	1.69	0.090	-	-	-	-
ACEs -> TTE	0.49	0.32	0.10	1.57	0.120	-	-	-	-
**Indirect effect**									
ACEs -> TTE -> Depression	-	-	-	-	-	-	-	-	-
ACEs -> TTE -> Anxiety	-	-	-	-	-	-	-	-	-
ACEs -> TTE -> Stress	-	-	-	-	-	-	-	-	-
**Divorce**	-	-	-	-	-	19.26	11	0.98	0.05 [0.00–0.09]
**Direct effects**									
ACEs -> Depression	0.64	0.64	0.05	1.00	0.320	-	-	-	-
ACEs -> Anxiety	0.27	0.50	0.03	0.54	0.590	-	-	-	-
ACEs -> Stress	1.16	0.69	0.09	1.68	0.090	-	-	-	-
ACEs -> TTE	0.41	0.20	0.12	2.03	0.040*	-	-	-	-
**Indirect effects**									
ACEs -> TTE -> Depression	-	-	-	-	-	-	-	-	-
ACEs -> TTE -> Anxiety	-	-	-	-	-	-	-	-	-
ACEs -> TTE -> Stress	-	-	-	-	-	-	-	-	-
**Domestic violence**	-	-	-	-	-	13.16	11	0.97	0.05 [0.00–0.10]
**Direct effects**									
ACEs -> Depression	0.51	0.71	0.04	0.71	0.480	-	-	-	-
ACEs -> Anxiety	0.47	0.54	0.05	0.87	0.380	-	-	-	-
ACEs -> Stress	1.43	0.72	0.10	2.00	0.050*	-	-	-	-
ACEs -> TTE	0.70	0.21	0.19	3.35	0.001*	-	-	Fit	-
**Indirect effects**									
ACEs -> TTE -> Depression	-	-	-	-	-	-	-	-	-
ACEs -> TTE -> Anxiety	-	-	-	-	-	-	-	-	-
ACEs -> TTE -> Stress	0.64	0.23	0.05	2.80	0.005*	-	-	*R*^2^ = 0.23^b^	-
**Substance use**	-	-	-	-	-	20.89	11	0.97	0.05 [0.01–0.09]
**Direct effect**									
ACEs -> Depression	0.82	0.64	0.07	1.28	0.200	-	-	-	-
ACEs -> Anxiety	1.35	0.49	0.14	2.76	0.006*	-	-	-	-
ACEs -> Stress	2.08	0.69	0.16	3.00	0.003*	-	-	-	-
ACEs -> TTE	0.53	0.17	0.15	2.68	0.007*	-	-	Fit	-
**Indirect effect**									
ACEs -> TTE -> Depression	-		-	-	-	-	-	-	-
ACEs -> TTE -> Anxiety	0.27	0.13	0.03	2.08	0.04*	-	-	*R*^2^ = 0.23^a^	-
ACEs -> TTE -> Stress	0.46	0.20	0.04	2.29	0.02*	-	-	*R*^2^ = 0.23^a^	-
**Household mental illness**	-	-	-	-	-	21.36	11	0.97	0.06 [0.02–0.09]
**Direct effect**									
ACEs -> Depression	3.19	0.70	0.24	4.59	< 0.001*	-	-	-	-
ACEs -> Anxiety	2.15	0.55	0.21	3.90	< 0.001*	-	-	-	-
ACEs -> Stress	2.93	0.76	0.21	3.84	< 0.001*	-	-	-	-
ACEs -> TTE	0.96	0.23	0.36	4.11	< 0.001*	-	-	Fit	-
**Indirect effect**								-	-
ACEs -> TTE -> Depression	0.52	0.22	0.04	2.40	0.020*	-	-	*R*^2^ = 0.28^a^	-
ACEs -> TTE -> Anxiety	0.43	0.17	0.04	2.55	0.010*	-	-	*R*^2^ = 0.25^a^	-
ACEs -> TTE -> Stress	0.76	0.27	0.05	2.86	0.004*	-	-	*R*^2^ = 0.24^a^	-
**Household prison**	-	-	-	-	-	18.88	11	0.98	0.05 [0.00–0.08]
**Direct effect**									
ACEs -> Depression	0.54	0.70	0.04	0.78	0.430	-	-	-	-
ACEs -> Anxiety	1.13	0.58	0.11	2.03	0.040	-	-	-	-
ACEs -> Stress	0.32	0.79	0.02	0.41	0.680	-	-	-	-
ACEs -> TTE	0.30	0.21	0.08	1.43	0.150	-	-	-	-
**Indirect effect**									
ACEs -> TTE -> Depression	-	-	-	-	-	-	-	-	-
ACEs -> TTE -> Anxiety	-	-	-	-	-	-	-	-	-
ACEs -> TTE -> Stress	-	-	-	-	-	-	-	-	-

s.e., standard error; TLI, Tucker–Lewis Index; RMSEA, root mean square error of approximation; Cl, confidence interval; ACEs, adverse childhood events; TTE, total traumatic events.

* , indicates *p* < 0.05;

a , indicates partial mediation;

b , indicates full mediation.

**TABLE 4:** Model fit statistics for adult trauma as a mediator between individual ACEs and mental health.

Model	*χ*^2^ (*df*)	CFI	TLI	RMSEA [Cl]
Total ACEs	17.37 (11)	0.97	0.98	0.04 [0.00, 0.08]
Verbal abuse	19.90 (11)*	0.95	0.98	0.05 [0.01, 0.09]
Physical abuse	17.65 (11)	0.97	0.98	0.04 [0.00, 0.08]
Sexual abuse	15.11 (11)	0.98	0.99	0.04 [0.00, 0.07]
Emotional neglect	16.46 (11)	0.97	0.98	0.04 [0.00, 0.08]
Physical neglect	18.66 (11)	0.96	0.98	0.05 [0.00, 0.08]
Divorce	19.26 (11)	0.96	0.98	0.05 [0.00, 0.06]
Domestic violence	16.16 (11)	0.97	0.97	0.05 [0.00, 0.10]
Substance use	20.89 (11)	0.95	0.97	0.05 [0.01, 0.09]
Household mental Illness	21.36 (11)	0.95	0.97	0.06 [0.02, 0.09]
Household prison	18.88 (11)	0.96	0.98	0.05 [0.00, 0.08]

CFI, comparative fit index; TLI, Tucker–Lewis Index; RMSEA, root mean square error of approximation; Cl, confidence interval; ACEs; adverse childhood events.

## Discussion

This study of a clinical sample of mental health patients at two large public psychiatric facilities in Johannesburg examined how adult trauma events mediated the relationship between ACEs and depression, anxiety and stress. The findings of this study reflect the high rates of ACEs that have been found in other research in sub-Saharan Africa, with up to 83% experiencing emotional abuse, 64% physical abuse and 19% sexual abuse.^3^ Another study in Soweto found that out of a cohort of 1636 children in Soweto township just outside Johannesburg, 35% had four or more ACEs.^42^ Similarly, another highly reputable South African study found physical abuse and psychological trauma reported in two-thirds of the study sample.^28^ Our research therefore contributes to the existing literature denoting a high rate of ACEs in South Africa.^9,43,44^

While other mediation studies in South Africa have examined variables linked to ACEs and mental health, none have examined the pathway of complex trauma over a patient’s life course to see the impact multiple traumatic events have over time. One mediation study looked at how childhood trauma is mediated by poor coping abilities, thus leading to depression and other psychiatric outcomes, with findings indicating that the ability to cope is a key factor in whether or not childhood trauma leads to mental illness in adulthood.^10^ Another mediation study similarly looked at childhood trauma and mental health outcomes such as depression among women with human immunodeficiency virus (HIV).^45^ This same study examined trauma as a mediator between depression and resilience, suggesting that resilience may be a protective factor against developing depression for women who experienced trauma. However, both of these studies focused on trauma specifically during the period of childhood.

As noted in Manyema et al.,^46^ the relationship between ACEs and adult stress is complicated, as it provides some evidence that adult trauma is a key mediator of childhood trauma when examining mental illness in an adult clinical population. Expanding on their findings using hierarchical regressions – which found both a direct effect of ACEs on psychological distress and an attenuation of that effect in the presence of adult stress – the current study used path analysis to show that, in our sample, adult traumatic events mediated the effects of ACEs on mental health outcomes. The results in the current study provide nuance to the findings of Manyema et al.^46^ as significant relationships were found with certain ACEs and not others. For example, ACEs such as experiencing physical abuse or physical neglect as a child had a very weak relationship to adult mental distress. However, experiencing emotional neglect or verbal abuse as a child had a very strong relationship to mental illness in adulthood across the board. This indicates that ACEs affecting more social and emotional factors had larger impacts on adult mental distress over time compared with physical factors and should be further explored in future research.

In addition to the direct effects on adult mental distress, adult traumatic events were found to partially mediate the association between ACEs, including verbal abuse, emotional neglect, mental illness in the household as a child, and substance use in the household as a child, and either all or some of the mental health outcome variables. Adult traumatic events fully mediated the associations between experiencing domestic violence in childhood or child sexual abuse and either all or some of the outcome variables. These findings support earlier research suggesting that the effect of ACEs is attenuated in the presence of adult adversity,^46,47,48^ although those studies explored ACEs and adult trauma through hierarchical regression, not mediational analysis. These findings therefore also expand on previous research by elucidating the association between ACEs and adult trauma; with the exception of physical abuse, physical neglect, and having a household member in prison during childhood, all other ACEs were significantly associated with the number of traumatic events participants experienced as adults, thereby supporting our hypothesis of adult traumatic events as a mediator. A recent study similarly found adult adversity to mediate the relationship between ACEs and poor adult mental health in an American non-clinical sample, although this study did not disaggregate ACEs.^49^ This analysis therefore highlights strong pathways in which psychiatric symptoms in adulthood follow a trajectory leading from traumatic experiences as a child to traumatic experiences as an adult and ultimately poor mental health.

This study also highlighted why it is important to research factors related to adult traumatic experiences. First and foremost is poverty, which is widespread in many sub-Saharan countries. Poverty is associated with increased rates of abuse, as poor households are often overstressed.^28,50^ Many of these adult traumatic experiences primarily affect women, as several studies have found that over half of women experience physical and/or sexual abuse as adults.^10,51,52^ Physical abuse by a partner is often the most common form of violence experienced by women in South Africa.^21,53^ Repeated trauma exposure – both in childhood and adulthood – can therefore result in more severe symptoms of psychopathology.^23^

Lastly, this study found a significant relationship between alcohol use and adult traumatic experiences. Similarly, other studies in South Africa which examined 560 women in a township outside Cape Town found that alcohol use has a strong relationship to both trauma and mental illness in this population.^54^ Alcohol use continues to be a public health problem in South Africa, along with other forms of substance abuse, which also have been found to adversely impact mental health outcomes.^55,56,57^ While the healthcare system is currently grappling with how to address these syndemic problems, only 5% of the public health budget is currently spent on mental health in South Africa, and the overburdened public sector – which services 84% of the population – is severely underfunded.^11^ This was compounded during the coronavirus disease 2019 (COVID-19) pandemic, which not only stretched already struggling public health services but also saw large increases in psychological illness among the population.^58^ Perhaps a simple way to increase screening for ACEs among patients in South Africa would be to train lay community health workers to administer the scale to patients in public health settings.

While this study provides interesting initial findings for future research on mental illness in adulthood, the results should be interpreted with caution as the study has limitations. Data were collected with a clinical population of adults, and therefore the findings may not be generalisable to the broader population. However, given the high rates of trauma and mental illness in South Africa paired with low rates of receiving mental health support, it is possible that these findings would be applicable across South Africa, and future research should explore this connection. Another limitation is the relatively low sample size in comparison with the number of variables and models. Additionally, this study collected data via retrospective self-reporting, which has been found to affect participant responses.^59^ Finally, we were not able to disaggregate the type and severity of adult trauma for this study because of sample size and therefore used the total number of events as a mediator. Future studies should further explore connections between specific traumatic events in adulthood and specific ACEs to further elucidate pathways which can be targeted for clinical support.

## Conclusion

Traumatic events in both childhood and adulthood are closely linked and represent a clear pathway to experiences of mental distress for adults. The high rates of depression, anxiety and stress in South Africa speak to the need to better understand the origins of major mental illness in the country as a precursor to developing methods to support the recovery of patients experiencing mental distress. The results of this study further that aim by improving our understanding of how the connections between specific forms of childhood adversity and adult trauma impact well-being, particularly in understudied regions of the world such as sub-Saharan Africa. While preventing the occurrence of ACEs is an important objective, so too is comprehending how to interrupt the chain linking ACEs and poor health outcomes. Lastly, this study also helps to identify which types of ACEs are most influential for adult trauma and mental health risks, emphasising how specific ACEs affecting social and emotional factors may impact adult mental distress more than physical forms of abuse.
